# ACUTE CHOLECYSTITIS IN HIGH-RISK PATIENTS. SURGICAL, RADIOLOGICAL, OR ENDOSCOPIC TREATMENT? BRAZILIAN COLLEGE OF DIGESTIVE SURGERY POSITION PAPER

**DOI:** 10.1590/0102-672020230031e1749

**Published:** 2023-09-15

**Authors:** Júlio Cezar Uili COELHO, Marco Aurélio Raeder da COSTA, Marcelo ENNE, Orlando Jorge Martins TORRES, Wellington ANDRAUS, Antonio Carlos Ligocki CAMPOS

**Affiliations:** 1 Universidade Federal do Paraná, Department of Surgery – Curitiba (PR), Brazil;; 2 Hospital Federal Ipanema – Rio de Janeiro (RJ), Brazil; 3 Hospital Samaritano – Rio de Janeiro (RJ), Brazil; 4 Universidade Federal do Maranhão, Department of Surgery – São Luís (MA), Brazil; 5 Universidade de São Paulo, Department of Gastroenterology, São Paulo (SP), Brazil

**Keywords:** Gallbladder, Acute cholecystitis, Cholecystectomy, Cholecystostomy, Laparoscopic drainage, Endoscopic drainage, Vesícula biliar, Colecistite aguda, Colecistectomia, Colecistostomia, Drenagem, Laparoscopia, Drenagem endoscópica

## Abstract

Acute cholecystitis (AC) is an acute inflammatory process of the gallbladder that may be associated with potentially severe complications, such as empyema, gangrene, perforation of the gallbladder, and sepsis. The gold standard treatment for AC is laparoscopic cholecystectomy. However, for a small group of AC patients, the risk of laparoscopic cholecystectomy can be very high, mainly in the elderly with associated severe diseases. In these critically ill patients, percutaneous cholecystostomy or endoscopic ultrasound gallbladder drainage may be a temporary therapeutic option, a bridge to cholecystectomy. The objective of this Brazilian College of Digestive Surgery Position Paper is to present new advances in AC treatment in high-risk surgical patients to help surgeons, endoscopists, and physicians select the best treatment for their patients. The effectiveness, safety, advantages, disadvantages, and outcomes of each procedure are discussed. The main conclusions are: a) AC patients with elevated surgical risk must be preferably treated in tertiary hospitals where surgical, radiological, and endoscopic expertise and resources are available; b) The optimal treatment modality for high-surgical-risk patients should be individualized based on clinical conditions and available expertise; c) Laparoscopic cholecystectomy remains an excellent option of treatment, mainly in hospitals in which percutaneous or endoscopic gallbladder drainage is not available; d) Percutaneous cholecystostomy and endoscopic gallbladder drainage should be performed only in well-equipped hospitals with experienced interventional radiologist and/or endoscopist; e) Cholecystostomy catheter should be removed after resolution of AC. However, in patients who have no clinical condition to undergo cholecystectomy, the catheter may be maintained for a prolonged period or even definitively; f) If the cholecystostomy catheter is maintained for a long period of time several complications may occur, such as bleeding, bile leakage, obstruction, pain at the insertion site, accidental removal of the catheter, and recurrent AC; g) The ideal waiting time between cholecystostomy and cholecystectomy has not yet been established and ranges from immediately after clinical improvement to months. h) Long waiting periods between cholecystostomy and cholecystectomy may be associated with new episodes of acute cholecystitis, multiple hospital readmissions, and increased costs. Finally, when selecting the best treatment option other aspects should also be considered, such as costs, procedures available at the medical center, and the patient’s desire. The patient and his family should be fully informed about all treatment options, so they can help making the final decision.

## RESUMO

A colecistite aguda (CA) é um processo inflamatório agudo da vesícula biliar que pode estar associado a complicações potencialmente graves, como empiema, gangrena, perfuração da vesícula biliar e sepse. O tratamento padrão para a CA é a colecistectomia laparoscópica. No entanto, para um pequeno grupo de pacientes com CA, o risco de colecistectomia laparoscópica pode ser muito alto, principalmente em idosos com doenças graves associadas. Nestes pacientes críticos, a colecistectomia percutânea ou a drenagem endoscópica da vesícula biliar guiada por ultrassom podem ser uma opção terapêutica temporária, como ponte para a colecistectomia. O objetivo deste artigo de posicionamento do Colégio Brasileiro de Cirurgia Digestiva é apresentar novos avanços no tratamento da CA em pacientes cirúrgicos de alto risco, para auxiliar cirurgiões, endoscopistas e clínicos a selecionar o melhor tratamento para os seus pacientes. A eficácia, segurança, vantagens, desvantagens e resultados de cada procedimento são discutidos. As principais conclusões são: a) Pacientes com CA e risco cirúrgico elevado devem ser tratados preferencialmente em hospitais terciários onde a experiência e os recursos cirúrgicos, radiológicos e endoscópicos estão disponíveis. b) A modalidade de tratamento ideal para pacientes com elevado risco cirúrgico, deve ser individualizada, com base nas condições clínicas e na experiência disponível. c) A colecistectomia laparoscópica continua sendo uma excelente opção de tratamento, principalmente em hospitais em que a drenagem da vesícula biliar percutânea ou endoscópica não está disponível. d) A colecistostomia percutânea e a drenagem endoscópica da vesícula biliar devem ser realizadas apenas em hospitais bem equipados e com radiologista intervencionista e/ou endoscopista experientes. e) O cateter de colecistostomia deve ser removido após a resolução da CA. No entanto, em pacientes que não têm condição clínica para realizar colecistectomia, o cateter pode ser mantido por um período prolongado ou mesmo definitivamente. f) Se o cateter de colecistostomia for mantido por longo período de tempo podem ocorrer várias complicações, como sangramento, fístula biliar, obstrução, dor no local de inserção, remoção acidental do cateter e CA recorrente. g) O tempo de espera ideal entre a colecistostomia e a colecistectomia ainda não foi estabelecido, e vai desde imediatamente após a melhoria clínica, até meses após. h) Longos períodos de espera entre colecistostomia e colecistectomia podem estar associados a novos episódios de CA, múltiplas readmissões hospitalares e aumento dos custos. Finalmente, ao selecionar a melhor opção de tratamento, outros aspectos também devem ser considerados, como custos, disponibilidade dos procedimentos no centro médico e o desejo do paciente. O paciente e sua família devem ser completamente informados sobre todas as opções de tratamento, para que possam ajudar a tomar a decisão final.

## SUMMARY OF THE MAIN RECOMMENDATIONS

- Patients with acute cholecystitis and high surgical risk must be preferably treated in tertiary hospitals where surgical, radiological, and endoscopic expertise and resources are available.
- The optimal treatment modality for patients with acute cholecystitis and high surgical risk should be individualized based on patient conditions and available expertise.
- Laparoscopic cholecystectomy remains an excellent option of treatment, mainly in hospitals in which percutaneous or endoscopic gallbladder drainage is not available.
- Percutaneous cholecystostomy and endoscopic gallbladder drainage should be performed only in well-equipped hospitals with experienced interventional radiologist and/or endoscopist.
- Cholecystostomy catheter should be removed after resolution of acute cholecystitis. However, in patients who have no clinical condition to undergo cholecystectomy, the catheter may be maintained for a prolonged period or even definitively.
- If the cholecystostomy catheter is maintained for an extended period of time several complications may occur, such as bleeding, bile leakage, obstruction, pain at the insertion site, accidental removal of the catheter, and recurrent acute cholecystitis.
- The ideal waiting time between cholecystostomy and cholecystectomy has not yet been established and ranges from immediately after clinical improvement to months.
- Long waiting periods between cholecystostomy and cholecystectomy may be associated with new episodes of acute cholecystitis, multiple hospital readmissions, and increased costs.
- When both percutaneous cholecystostomy and endoscopic gallbladder drainage are available in the hospital, the preferred choice should be endoscopic gallbladder drainage because it has a lower complication rate.
- There is no consensus on how long an expandable metal stent should be maintained following endoscopic gallbladder drainage.
- Gallbladder drainage through transpapillary approach should be reserved only for patients in whom it is not possible to employ the endoscopic procedure once this last method has a higher rate of technical and clinical success.

## INTRODUCTION

Acute cholecystitis (AC) is an inflammatory process of the gallbladder that may be associated with potentially severe complications, such as empyema, gangrene, perforation of the gallbladder, and sepsis^6,15,37^. It is caused by gallstone obstruction of the cystic duct in 90 to 95% of cases^37^. AC is the most common complication of gallstone disease. Approximately 20 to 30% of patients with symptomatic cholelithiasis will develop AC at some point in their lives, usually after repeated episodes of biliary colic^34^. However, AC may be the first clinical manifestation of gallstone. It is one of the most common surgical emergencies. In the United States more than 200,000 individuals are diagnosed with AC annually, justifying the great interest in this subject^15^.

In approximately 5 to 10% of AC patients, no gallstone is found, which is referred to as acalculous AC^2^. The etiology of the gallbladder acute inflammation in these patients is multifactorial and includes a multitude of critical illnessess, such as diabetes, human immunodeficiency virus (HIV), cytomegalovirus infection (CMV), trauma, and prolonged total parenteral nutrition^2,15^. Although acute acalculous cholecystitis is an uncommon disease, its incidence is increasing, and its mortality far exceeds that of acute calculous cholecystitis^15^.

The gold standard treatment for AC consists of early laparoscopic cholecystectomy, preferably within the first 24 hours of hospital admission^4,15,25^. Cholecystectomy can be successfully performed laparoscopically in almost all AC patients^4^. However, the complication rate and conversion to open surgery is higher than that of laparoscopic cholecystectomy performed for chronic cholecystitis.

For a small group of AC patients, the risk of laparoscopic cholecystectomy can be very high, mainly in the elderly with associated severe diseases^5^. In these critically ill patients, percutaneous cholecystostomy or endoscopic ultrasound gallbladder drainage may be a temporary therapeutic option, a bridge to cholecystectomy. After adequate recovery of the patient, the definitive treatment — laparoscopic cholecystectomy — may be performed. However, many patients will remain at high surgical risk due to advanced age and comorbidities, and a cholecystectomy may not be performed^3,13,14^. In this condition, AC may recur and stent complications are frequently observed.

### What is the objective of this paper?

The objective herein is to present new advances in AC treatment in high-risk surgical patients and to help surgeons, endoscopists, and physicians select the best treatment for their patients. The effectiveness, safety, advantages, disadvantages, and outcomes of each procedure are discussed. Procedure availability and cost are important factors in decision-making process in any country, mainly in a large developing country like Brazil. These relevant aspects were also considered.

### What does high surgical risk mean?

There are no widely accepted criteria to determine which patient with AC has high surgical risk and should avoid a cholecystectomy. Most surgeons make the decision based on a combination of the patients’ age and the presence of associated severe comorbidities^14^.

Complications of AC surgery are higher in the elderly. Age over 80 is an independent risk factor for higher morbidity and mortality after cholecystectomy^5,15,17^. The intensity of gallbladder inflammation and the presence of complications, such as gallbladder empyema and gangrene, should also be considered. Previous upper quadrant abdominal operations may also increase the difficulty of dissection and identification of anatomical structures due to intense and firm fibrosis.

In general, radiological (percutaneous cholecystostomy) or endoscopic treatment is indicated in critically ill patients, mainly over 80 years of age, in whose risk of cholecystectomy is very high^12,13,45^. The main comorbidities that greatly increase surgical risk are heart disease, advanced lung disease, liver cirrhosis, immunosuppression, advanced malignancy, coagulopathy, and sepsis. Some authors employ classification systems, such as the ASA-PS (American Society of Anesthesiologists — Physical Status) classification, the APACHE II (Acute Physiology Assessment and Chronic Health Evaluation II), and the Tokyo Guidelines of 2018 for diagnostic criteria and severity grading of AC to assess surgical risk and to select patients for radiological or endoscopic procedure^29,47^.

Patients with temporary severe immunodeficiency — after chemotherapy or bone marrow transplantation — who develop AC may also be considered for radiological or endoscopic treatment, even if young.

Another indication for radiological or endoscopic procedure is the patient with hepatopancreatobiliary neoplasia who underwent the placement of a biliary prosthesis as a treatment for biliary obstruction. This patient may develop AC due to the obstruction of cystic duct by the biliary prosthesis or by neoplasia in approximately 2 to 10% of cases. In these conditions, some oncologists prefer to indicate radiological or endoscopic treatment rather than perform laparoscopic cholecystectomy to avoid interrupting chemotherapy and/or radiotherapy.

### When is cholecystectomy indicated for patients with high surgical risk?

There is no consensus on which is the best treatment for high-risk patients with AC^2,3,10^. Laparoscopic cholecystectomy remains an excellent option of treatment, mainly in hospitals in which percutaneous or endoscopic gallbladder drainage is not available^8,9,15,16^.

Emergency laparoscopic cholecystectomy is the gold standard treatment for almost all patients with AC^13,18^. However, in critically ill patients or the elderly with severe comorbidities, the surgical risk of cholecystectomy can be significant and may result in mortality superior to 10%^46^.

In these patients, less invasive procedures, such as radiological or endoscopic treatment of AC could be an attractive alternative. However, radiological and endoscopic procedures are not definitive treatments since the gallbladder is not removed. When computed the risks of additional cholecystectomy that should be performed later after the patient recovery, the combined mortality, morbidity, readmissions, and hospital stay are lower for the cholecystectomy. If cholecystectomy is delayed after percutaneous or endoscopic gallbladder drainage, recurrent cholecystitis and complications associated with the catheter are significant^46^. Other aspects should also be considered, such as costs, procedures availability at the medical center, and the patient’s desire. The patient and his family should be fully informed about all treatment options, so they can make the final decision.

Emphysematous cholecystitis is a rare form of acute cholecystitis in which gas-forming bacteria infect the gallbladder wall. It is associated with high mortality rate. In this case, cholecystectomy is the treatment of choice because of the high risk of gallbladder necrosis and perforation.

### When may percutaneous cholecystostomy or endoscopic ultrasound-guided gallbladder drainage be indicated for patients with acute cholecystitis?

Percutaneous cholecystostomy (PC) and endoscopic ultrasound-guided gallbladder drainage (EUS-GBD) are reserved for critically ill patients with severe AC, mainly the elderly or patients with severe immunodeficiency who require a limited procedure both in terms of extension and time^7,29,30,32,42^. It is important to emphasize that due to the severity of patient´s disease, these procedures should be performed only in hospitals with experienced and well-trained interventional radiologist and endoscopist.

The 2022 Guideline of the European Society of Gastrointestinal Endoscopy recommends that EUS-GBD should be favored over PC where both techniques are available, owing to the lower rates of adverse events and the need for re-intervention following EUS-GBD^44^. Although endoscopic drainage of the gallbladder is considered a superior procedure, it is available only in a few tertiary hospitals. Therefore, PC is still the most used procedure to drain the gallbladder in AC patients at high surgical risk.

It is important to emphasize that PC and EUS-GBD are not definitive treatments for AC, and a significant number of patients will need laparoscopic cholecystectomy after adequate recovery.

### What are the important technical aspects of percutaneous cholecystostomy?

PC was used for the first time by Radder^38^ in 1980 for the treatment of AC in critically ill patients. PC should be performed by an experienced radiologist in a properly equipped radiologic room^21,22^. Access can be done through the transhepatic or transabdominal route. The transhepatic route is preferred by the majority because it is associated with lower rate of bile leakage and, consequently, of biliary peritonitis^15,22,25^.

The procedure is usually performed under local anesthesia, with the patient under conscious sedation. The Seldinger method is used. The gallbladder is punctured with an appropriate needle guided by ultrasonography. After insertion of the guidewire, the path is dilated, and an 8 or 10-French pigtail PC catheter is inserted into the gallbladder guided by fluoroscopy. Samples of the gallbladder contents are sent for microbiological culture and Gram staining. The catheter is fixed to the skin with sutures and connected to a bedside bag.

### What is the outcome of percutaneous cholecystostomy?

PC resolves the acute inflammatory process of the gallbladder and controls biliary infection in about 90% of AC patients. In a systematic review of 53 studies with 1918 patients, the overall 30-day mortality was 15.4% and was mainly related to patient’s associated severe comorbidities^15,25^.

More recently, some authors questioned the advantages of PC in the treatment of AC^15,40^. In a study with data obtained from the United States National Database of 358,624 elderly patients (≥65 years) with AC and high surgical risk, it was observed that patients submitted to PC had less satisfactory results than those submitted to open or laparoscopic cholecystectomy^40^. It had higher mortality, higher complication rate, and an increased rate of hospital readmission than open or laparoscopic cholecystectomy^36^.

In a systematic review and meta-analysis, Hemerly etal.^20^ reported that EUS-GBD was superior to PC in terms of safety profile, recurrent cholecystitis, and hospital readmission rates in the management of AC patients who were suboptimal candidates for cholecystectomy. Other recent studies confirmed that PC results were inferior to those for cholecystectomy^17,19^. These studies showed that PC — a simple drainage of the gallbladder — does not solve the etiological factor of AC and question whether this simple procedure has a true benefit in reducing morbidity, mortality, and hospital costs. Additional prospective and randomized studies are needed to determine the real advantages of PC^19^.

### What are the complications of percutaneous cholecystostomy?

PC can be associated with several complications, both early and late. The main ones are the catheter displacement or obstruction, hemorrhage, hepatic hematoma, hemobilia, biliary peritonitis, intestinal perforation, sepsis, abdominal abscess, and pneumothorax.

The PC mortality is generally <3%, an acceptable rate considering the high severity in most patients in which the procedure is performed^15^. These rates do not include the mortality that may occur later in the follow-up of patients due to laparoscopic cholecystectomy, replacement, and/or the need for an additional cholecystostomy.

### What are the contraindications of percutaneous cholecystostomy?

PC does not have an absolute contraindication. However, it should be avoided in the presence of ascites, mainly in patients with advanced liver cirrhosis. Ascites may predispose to bile leakage through the stent entrance into the liver. Nevertheless, PC may be performed after ascites drainage and administration of diuretics and albumin. Coagulopathy should be adequately corrected before the procedure^11^. Another relative contraindication for PC is gallbladder cancer due to the possibility of neoplastic dissemination^24^.

### When should the catheter be removed after percutaneous cholecystostomy?

The cholecystostomy catheter should generally be removed after AC resolution. Subsequently, a cholecystectomy should be performed. However, in patients with no clinical conditions to undergo cholecystectomy, the catheter may be maintained for a prolonged period or even definitively^3,18^. Many patients will need to have the catheter changed in case of obstruction or be reintroduced in case of accidental removal.

Some authors observed that the catheter is removed even in patients who will not be subjected to cholecystectomy. In this condition, AC may recur. The recurrence of AC after catheter removal ranges from 4 to 22% in the literature^13,15^.

Based on the information collected from the United States National Database from January to November 2013, Pavurala etal.^36^ reported that of 3,167 patients who underwent cholecystostomy, only 1,196 (37.8%) underwent cholecystectomy within a period of up to one year. The remaining 1,971 (62.2%) stayed with the cholecystostomy tube during this period. The study with the entire United States population demonstrated that almost two-thirds of the patients submitted to supposedly temporary percutaneous cholecystostomy will remain with the catheter for a prolonged time or possibly permanently^36^.

### Should all patients who underwent percutaneous cholecystostomy be subjected to cholecystectomy?

Initially, PC was thought to be a bridge procedure, allowing a laparoscopic cholecystectomy with lower surgical risk after the regression of the gallbladder inflammatory process and adequate recovery of the patient. However, many patients who undergo PC will never be subjected to cholecystectomy due to advanced age and/or severe associated diseases^26,31^. In this population, a permanent percutaneous drain may resolve.

The catheter has several notable disadvantages including the risk of bleeding, bile leakage, obstruction, pain at the insertion site, and accidental removal of the catheter^46^. In addition, AC recurs in 22 to 47% of patients with permanent catheter^13^. Therefore, the recurrence of catheter exchange in case of obstruction and its reintroduction in AC patients is frequent^15^. As a result, percutaneous drainage is associated with a decrease in quality of life^17^.

However, in patients whose clinical conditions remain critical and cannot support any operation, the catheter can be removed after AC resolution or maintained permanently.

### When should cholecystectomy be performed in patients who underwent percutaneous cholecystostomy?

The ideal waiting period between cholecystostomy and cholecystectomy has not yet been established; it ranges from immediately after clinical improvement to over 8 weeks^13,26,39,46^. With the information obtained from the New York State Database of the United States of 9,728 patients who underwent cholecystostomy between 2000 and 2012, it was found that patients who underwent early cholecystectomy (≤8 weeks) had a higher complication rate and longer hospitalization than patients with late cholecystectomy (＞8 weeks)^17,42^. However, very long waiting periods may be associated with new episodes of AC, multiple hospital readmissions, and increased costs^13,36,46^.

Performing laparoscopic cholecystectomy after AC resolution by percutaneous gallbladder drainage is a technically more difficult procedure and is associated with a higher percentage of complications, including bile duct injury, given the intense adhesions resulting from the gallbladder drainage catheter and the previous episode of AC. The incidence of conversion to open surgery is also higher^36,46^. Most patients will not be submitted to laparoscopic cholecystectomy after cholecystostomy due to high surgical risk, especially in the elderly over 80 years of age or in patients with severe comorbidities.

### What are the important technical aspects of endoscopic ultrasound-guided gallbladder drainage?

EUS-GBD was first described by Baron and Topazian^1^ in 2007 using transmural placement of double pigtail biliary stents to communicate the gallbladder with the duodenum in a patient with AC. The patient had unresectable hilar cholangiocarcinoma previously treated with the placement of palliative bilateral metal biliary stent.

The procedure consists of accessing the gallbladder from the stomach or duodenum lumen and placing a stent within the gallbladder to communicate with the gastrointestinal tract, avoiding an external drain. Preprocedural imaging, either computed tomography or magnetic resonance, is essential in determining if EUS-GBD is feasible and for selecting the puncture site.

Under general anesthesia, a linear echoendoscope is inserted into the stomach or duodenum and the gallbladder is identified endosonographically.

EUS-GBD is performed either by conventional or direct methods^33,41^. In the conventional procedure, the stent is placed in the gallbladder through a guidewire inserted through a needle. In the direct, the stent is directly inserted into the gallbladder without prior needle insertion.

The proximal end of the stent is placed in the gallbladder and the distal end, in the gastrointestinal lumen. Bile sample is aspirated and sent to bacterial culture and Gram staining to guide antibiotic therapy.

The type of stent used is at the discretion of the endoscopist, based on several factors such as the fistulous tract length, the gallbladder wall thickness, and the gallbladder lumen and stone sizes^33,41^. The cautery-enhanced lumen-apposing metal stent (LAMS) is the most employed. After adequate placement of the stent, the gallbladder is completely emptied by suction and irrigation.

### What is the outcome of endoscopic ultrasound-guided gallbladder drainage?

EUS-GBD has emerged as a safe, efficacious, and minimally invasive option to treat AC in patients at high surgical risk. Several studies including a recent trial, comparing the efficacy of EUS-GBD and PC-GBD, have shown similar clinical success in both groups. However, the EUS-GBD group had lower rates of adverse events, unplanned readmissions, reinterventions, post- procedure pain, and length of hospital stay^31,33,44^.

With increasing evidence on the efficacy of endoscopic gallbladder drainage, the 2022 Guideline of the European Society of Gastrointestinal Endoscopy recommended that EUS-GBD should be preferred over PC-GBD due to lower rates of adverse events and reintervention^44^.

As a limitation, EUS-GBD is a technically difficult procedure associated with a steeper learning curve. Presently, it is available only in a few large medical centers. Anatomy modified by surgical operations, such as that observed after Roux-en-Y gastric bypass, may be technically challenging to perform with EUS-GBD^40^.

### What are the complications of endoscopic ultrasound-guided gallbladder drainage?

Technical and clinical success rates of EUS-GBD are higher than 90%, with a 12% adverse events rate. The main complications are acute pancreatitis, AC recurrence, stent migration, or catheter dislodgement^43^. Stent occlusion by food particles can lead to recurrent cholecystitis and has been observed more commonly when the drainage site is the stomach rather than the duodenum.

Although EUS-GBD and PC have similar technical and clinical success rates, resolution time of symptoms, hospital stay, need for repeat interventions, and morbidity are lower in the EUS-GBD group.

### What are the contra-indications of endoscopic ultrasound-guided gallbladder drainage?

EUS-GBD is contraindicated in patients with gallbladder necrosis or perforation and in those unable to tolerate sedation or hemodynamically unstable. A better option for these patients is to perform a PC which can be done under local anesthesia or as a bedside procedure.

### When should the stent be removed in patients who underwent endoscopic ultrasound-guided gallbladder drainage?

At present, there is no agreement on when to remove an expandable metal stent following EUS-GBD. However, there are concerns about delayed bleeding, perforation, or obstruction. Many centers prefer to replace the stent after tract maturation with plastic pigtail stents to allow for continuous drainage without concern about recurrent cholecystitis. Additional studies are needed to determine the optimal time interval to change or remove the stent.

### Is there still indication for gallbladder drainage through the transpapillary approach?

Gallbladder drainage through the endoscopic transpapillary approach (ET-GBD) should be reserved for patients in whom it is not possible to perform EUS-GBD once this last method has a higher rate of technical and clinical success.

ET-GBD was described by Kozarek etal.^27^ in 1984 as an alternative method of internal gallbladder drainage in AC patients at high surgical risk. The procedure is performed through the major duodenal papilla during endoscopic retrograde cholangiopancreatography (ERCP). After the cannulation of cystic duct, a guidewire is inserted through this duct into the gallbladder. Then, a transpapillary double pigtail plastic stent is placed into the gallbladder to allow drainage and irrigation^23^.

ET-GBD is a technically challenging procedure. The impossibility of completely cannulating the cystic duct in AC patients is significant due to tortuosity and inflammation of the duct and its obstruction by stones. Significant complications may occur, such as acute pancreatitis, perforation, and bleeding.

One of the largest studies comparing ET-GBD to EUS-GBD in patients unfit for cholecystectomy was performed by Oh etal.^35^ This study retrospectively assessed 172 patients with 76 in the ET-GBD group and 96 in the EUS-GBD group. The success rate was 83.3% for ET-GBD and 100% for EUS-GBD. The rate of adverse events was similar between ET-GBD and EUS-GBD at 9.4% and 7.2%, respectively. Recurrent cholecystitis or cholangitis occurred at a higher rate in the ET-GBD group (17.4%) than in the EUS-GBD group (3.9%).

In a systematic review and meta-analysis, Krishnamoorthi etal.^28^ reported that EUS-GBD had a higher rate of technical and clinical success and a lower rate of recurrent AC compared to ET-GBD. The rates of overall adverse events were similar. Hence, EUS-GBD was preferable to ET-GBD for the endoscopic management of AC in high-risk patients.

The 2022 Guideline of the European Society of Gastrointestinal Endoscopy recommends EUS-GBD over ET-GBD, due to the suboptimal technical efficacy of transpapillary gallbladder drainage.
